# Challenges and Adaptations for Providing Smoking Cessation for Patients with Cancer across Canada during the COVID-19 Pandemic

**DOI:** 10.3390/curroncol29040184

**Published:** 2022-03-24

**Authors:** Graham W. Warren, Caroline Silverman, Michelle Halligan

**Affiliations:** 1 Department of Radiation Oncology, Medical University of South Carolina (MUSC), Charleston, SC 29425, USA; warrengw@musc.edu; 2 Department of Cell and Molecular Pharmacology and Experimental Research, Medical University of South Carolina (MUSC), Charleston, SC 29425, USA; 3 Canadian Partnership Against Cancer, Toronto, ON M5H 1J8, Canada; caroline.silverman@partnershipagainstcancer.ca

**Keywords:** smoking cessation, quality care, implementing smoking cessation

## Abstract

Smoking cessation after a cancer diagnosis can improve health outcomes, but the Coronavirus disease 2019 (COVID-19) pandemic significantly altered healthcare patterns and strained resources, including for smoking cessation support for cancer patients. A Network that included all 13 provinces and territories (jurisdictions) in Canada received funding and coordinated support from a national organization to implement access to smoking cessation support in cancer care between 2016 and 2021, including throughout the COVID-19 pandemic. Descriptive analyses of meetings between the organization and jurisdictions between March of 2020 and August of 2021 demonstrated that all jurisdictions reported disruptions of existing smoking cessation approaches. Common challenges include staff redeployment, inability to deliver support in person, disruptions in travel, and loss of connections with other clinical resources. Common adaptations included budget and workflow adjustments, transition to virtual approaches, partnering with other community resources, and coupling awareness of the harms of smoking and COVID-19. All jurisdictions reported adaptations that maintained or improved access to smoking cessation services. Collectively, data suggest coordinated national efforts to address smoking cessation in cancer care could be crucial to maintaining access during an international healthcare crisis.

## 1. Introduction

The Coronavirus disease 2019 (COVID-19) pandemic has severely disrupted medical care patterns worldwide. Patients attended fewer medical visits due to COVID-19 concerns and healthcare systems modified medical treatment protocols to accommodate COVID-19 related risks [1,2,3]. Many health systems rapidly transitioned from in-person care to virtual patient visits [4]. In oncology care, many patients experienced reduced access to care as health systems considered methods to reduce risk of exposure while continuing to provide adequate care for cancer control [5,6]. Cancer screening was reduced, and it is expected that delays in subsequent care could lead to significant increases in cancer mortality over the coming decade [7,8,9,10]. However, as the COVID-19 pandemic continues to mature, efforts are increasingly focused on ensuring that patients and healthcare providers can access resources to deliver high-quality, evidence-based care [11].

Smoking is a proven risk factor for the development of many cancers and continued smoking after a cancer diagnosis is associated with increased overall mortality, cancer-related mortality, and added costs from cancer treatment [12,13,14,15]. Smoking cessation reduces risk for developing cancer and is further associated with improved survival, even after a cancer diagnosis [15]. Smoking cessation is now regarded as a core component of cancer care in many cancer organizations [16]. In the United States, the Cancer Center Cessation Initiative (C3I), sponsored by the National Cancer Institute (NCI), supports the development of evidence-based smoking cessation programs in 52 NCI-Designated Cancer Centers [17]. In Canada, the Canadian Partnership Against Cancer (“the Partnership”) supports the implementation of smoking cessation as a core component of cancer care across all provinces and territories [18]. These initiatives significantly increased access to evidence-based smoking cessation support for cancer patients prior to the COVID-19 pandemic. However, while a single institution’s experience has suggested that smoking cessation in cancer care can be sustained during the COVID-19 pandemic [19], there is little information about how COVID-19 has affected broad smoking cessation implementation efforts in cancer care. The purpose of this manuscript is to describe challenges and adaptations to providing access to smoking cessation support across Canada during the COVID-19 pandemic.

## 2. Materials and Methods

Starting in 2015, the Partnership began to support the development and implementation of evidence-based smoking cessation in cancer care in Canada, which included providing initial funding for nine provinces and territories (“jurisdictions”). In 2019, two- to three-year funding was provided to all 13 jurisdictions to implement smoking cessation approaches tailored to the resources and needs of each province or territory. Each jurisdiction was encouraged to develop or enhance approaches that would promote access and support to all cancer patients, including use of the 3A (Ask, Advise, Act) or 5A (Ask, Advise, Assess, Assist, Arrange) model for smoking cessation [16,20]. In Canada, traditional tobacco or sacred tobacco is used by some First Nations or Métis communities in ceremonial or sacred rituals for healing and purifying. For the purposes of this manuscript and associated discussion, smoking and smoking cessation are referring only to commercially produced combustible tobacco.

The Pan-Canadian Tobacco Cessation and Cancer Care Network (Network), first convened by the Partnership in 2017, consists of officials working in tobacco control and cancer programs representing each jurisdiction, and from the federal government. The Partnership provides Network members centralized support, including access to expertise in smoking cessation and logistical support to assist with program development and evaluation. Network meetings are convened by the Partnership twice annually. The Network further serves as an engaged collaborative where members share information, results, problems, solutions, and expertise.

Network activities were designed to support the implementation of smoking cessation activities across jurisdictions and were not designed as a research project. Network meetings and individual jurisdictional calls were recorded or documented with non-standardized notes, rather than through standardized questionnaires. Each individual jurisdictional call was focused on the specific accomplishments and needs of that jurisdiction. As an example, a jurisdiction that had not yet effectively implemented screening for smoking could be focused on improving those activities. A jurisdiction that had already achieved referrals to treatment programs could be focused on providing medications. Jurisdictional calls focused on tracking progress, assisting in problem solving, and sharing knowledge, including connecting jurisdictions to each other for experiential support.

The COVID-19 pandemic required shifting the Network meetings from in-person to online. The online meeting format was used in July 2020, January 2021, and June 2021. At the 2020 meeting, each jurisdiction was asked to provide specific examples of challenges and opportunities they experienced because of the COVID-19 pandemic, including their ability to implement or maintain core functions of counseling and pharmacotherapy for cancer patients. In addition, virtual calls were conducted approximately every two months and as needed between the Partnership and the project team for each jurisdiction. Prioritization was given to sharing the status of funded projects and implementation challenges posed by the COVID-19 pandemic and discussing strategies to help projects maintain or restore smoking cessation activities in cancer care within their jurisdiction. Meetings and jurisdictional calls were documented through unstructured, free-text notes by Partnership staff.

Data reported describe findings from a review of free-text notes collected during Network meetings, individual calls with jurisdictions, and jurisdictions’ self-assessed performance during the COVID-19 pandemic between March 2020 and August 2021. Descriptive data were extracted from meeting notes to examine challenges faced by jurisdictions and describe real-world adaptations over time. In addition, in March 2021, each jurisdiction provided the Partnership with self-reported information on performance metrics using the scale shown in Table 1, including the ability to deliver evidence-based care through behavioral counseling and pharmacotherapy.

## 3. Results

Though implementation of smoking cessation in cancer care settings progressed at different rates across jurisdictions between 2017 and 2020, several common program elements had been achieved by many of the jurisdictions by the outset of the COVID-19 pandemic in 2020, including:

- Structured screening and smoking cessation processes
- Incorporation of smoking cessation as a standard medical directive for cancer care
- Support and/or buy-in from administrative leadership, physicians, and other staff
- Ability to collect and analyze data
- Reporting of results to staff and leadership
- Partnerships with community resources
- Training and educational materials for clinicians and patients
- Development of feedback loops between smoking cessation services and cancer care
- Cross-jurisdictional collaborations to share information about addressing smoking across cancer care
- Efforts to engage at-risk populations and incorporate culturally competent support for Indigenous patients with cancer

At the first online Network meeting in July 2020, jurisdictions began reporting challenges and opportunities associated with the COVID-19 pandemic. Table 2 summarizes challenges and opportunities reported by jurisdictions between 2020 and 2021. All jurisdictions reported disruptions of existing smoking cessation approaches, with most noting the partial or full loss of one or more common program elements. The most frequent challenges reported by 83% of jurisdictions were the redeployment of staff to COVID-19-related activities or the inability to delivery smoking cessation in person. Changes in the ability to refer patients to community resources for smoking cessation support was also a common theme. Broader health systems issues were also described, including reductions in in-person patient visits at cancer centers, difficulty with distributing medications, disruptions in medical travel for patients and providers, loss of community resources, and changes in clinical and administrative staffing and infrastructure.

In response to challenges posed by the pandemic, jurisdictions reported unique opportunities to adapt so that they could continue to offer patients smoking cessation support (Table 2). Several reported a shift toward the use of virtual or phone-based approaches for smoking cessation counseling. Some reported partnering with other clinical groups and community resources to assist with smoking cessation, such as primary care physicians, pharmacists, and community behavioral health programs. Several jurisdictions also increased awareness for smoking cessation by coupling discussions about smoking cessation and risk reduction for COVID-19. When treatment services were interrupted, jurisdictions reported devoting more time to collecting or analyzing data and improving business plans for sustaining or enhancing existing smoking cessation services. Importantly, challenges related to a reduction in in-person appointments and medication distribution were viewed as opportunities by some jurisdictions to pilot offering phone-based care and free smoking cessation medications, improving delivery systems for sending medications to patients’ homes, and providing better long-term follow-up to patients. No jurisdiction reported an inability to adapt their smoking cessation approaches due to the COVID-19 pandemic.

Throughout the COVID-19 pandemic, jurisdictions were supported with financial and implementation support through the Partnership. Challenges and recovery adaptations were recorded at the bi-monthly meetings. Table 3 demonstrates shifting patterns of program reduction challenges and recovery adaptations. In most cases, challenges preceded adaptations by three to six months. However, some jurisdictions that had been less affected by the primary wave of COVID-19 early in 2020 reported challenges during successive COVID-19 waves, such as health system changes, staff losses, and difficulty with data collection. Recovery adaptations occurred rapidly, with a peak in adaptations beginning in the June to September 2020 timeframe. Nearly all jurisdictions required modifications to their funded-project budgets or workplans. Despite this, jurisdictions’ self-assessed performance on behavioral counseling and pharmacotherapy was the same or higher in March 2021 than it was in March 2020.

By August 2021, most jurisdictions reported that changes in staffing, leadership or support had stabilized or improved. Many programs had transitioned to refining delivery of smoking cessation, such as changing smoking assessments, using electronic medical records, improving data collection and analysis, and expanding education to clinicians and patients. However, concerns were raised about upcoming clinician retirements and physician shortages, restructuring of health services such as quitlines, and anticipated resurgence of COVID-19 waves. While some jurisdictions reported concerns about strained healthcare resources, such as views that clinicians were ‘just surviving’, there were parallel views that providing smoking cessation support had ‘strong physician engagement’ and helped reduce burden for clinicians. Among programs that were in a pre-implementation or early adoption phase, all were able to develop smoking cessation strategies within changing healthcare systems. Through August of 2021, all jurisdictions were able to adapt to challenges and maintain or improve self-reported assessments for counseling or pharmacotherapy.

## 4. Discussion

Data suggest that national efforts to address smoking by cancer patients were maintained during an international pandemic with the support of centralized coordination focused on assisting local resources to adapt to significant shifts in health care. Widespread disruptions experienced across most cancer care settings included staff redeployments and changes in non-smoking cessation-related care patterns, requiring shifting from in-person to virtual visits. Common opportunities and adaptations included increased use of virtual care, education about risks of smoking and COVID-19, and increased collaborations with other healthcare providers and community resources. While not explicitly stated, data suggest that jurisdictions adapted to loss of in-person counseling by advancing alternative cessation-related activities, such as developing innovative approaches to increase access to counseling or medications. Importantly, jurisdictions reported the ability to maintain or increase access to behavioral counseling and pharmacotherapy between 2020 and 2021.

There are limited data on how smoking cessation programs adapt to changes caused by COVID-19. Previously, a single institution supported as a part of C3I reported on the reach and effectiveness of implementing smoking cessation in cancer care prior to and during the COVID-19 pandemic [19]. Analyzing results between January of 2018 and December of 2020, investigators reported substantial increases in reach for patients receiving smoking cessation support from 3.2% before implementation to 48.4% in 2020. There was no change in reach during the pandemic and reach was maintained even during the height of the pandemic. Effectiveness, measured as quit rates among smoking patients, also improved prior to the pandemic. However, effectiveness decreased during the pandemic, possibly reflecting challenges in the ability for individual cancer patients to quit smoking during the pandemic. The ability to maintain reach during the pandemic parallels what was observed in Canadian jurisdictions supported by the Partnership. Collectively, these data suggest that access to evidence-based smoking cessation as a part of a national initiative can be maintained during a pandemic.

A common element among jurisdictions in Canada was the need to adjust smoking cessation in cancer care program budgets and workplans during the pandemic. As with any medical practice, business plans and financial support are critical to sustaining an intervention. Cancellation of hospital procedures resulted in rapid and significant changes in health system revenues [1,21,22,23]. Many hospitals and health systems faced layoffs, furloughs, decreased compensation plans, staff redeployment, and budget adjustments, resulting in immediate cancellation of non-essential activities and deployment support programs to assist with financial and resourcing stresses among health systems [1,2,3,21,22,23,24,25]. Loss or redeployment of staff and resources was commonly reported among jurisdictions. These losses could directly impact the ability to screen for smoking, provide treatment, assist with data collection or analysis, and report results to leadership for sustained buy-in. Transition to virtual visits further exacerbated the need to reconsider budgets and workplans. Facilitating practical changes in financial planning, workplans, timelines, and reporting were all useful in assisting individual jurisdictions adapt to rapid changes in healthcare delivery. Whereas no conclusions can be drawn about whether national coordination through the Partnership was critical to maintaining access to smoking cessation in cancer care, it is suggested that the tailored assistance and cross-jurisdictional knowledge exchange helped jurisdictions maintain focus on smoking cessation in cancer care and contributed to success across the country. Moreover, it is unlikely that any structured trial of this magnitude would be possible because events such as the COVID-19 pandemic are unplanned and would require rapid real-world adaptations specific to a disruptive event.

While these data are the first to report on how a national coordinated effort could assist with maintaining smoking cessation in cancer care during a pandemic, there are several limitations. Results report on real-world coordination through Network meetings and individual jurisdictional calls focused on jurisdictional needs and priorities. It is likely that data collected represent the most timely and concerning issues discussed at jurisdictional calls, and underreport the full spectrum of issues that would be captured through a standardized research format. However, it is unlikely that any structured national research instrument could be developed and administered within the rapidly developing changes in health care during the pandemic. Another limitation is that scoring of jurisdictions’ ability to deliver counseling and pharmacotherapy was self-reported and could not be verified by alternative means. These self-reported assessments may overestimate true performance among jurisdictions and no alternative verification method, such as patient-reported outcomes, were available across all jurisdictions for comparison. Best-practice alternatives, such as patient-level data or independent verification of smoking cessation program access, were not performed or available for comparison. Data cannot support conclusions about whether individual smoking cessation rates changed during the pandemic. However, data suggest that national coordinated efforts such as the Partnership and Network may help maintain access to smoking cessation support during a pandemic.

The worldwide COVID-19 pandemic could be viewed as a unique opportunity to rapidly accelerate changes in health care that better adapt to patient needs. Some jurisdictions reported that shifting to telephone-based smoking cessation counseling increased access for rural and remote populations and provided opportunities for improved long-term follow-up. Reviews of telehealth initiatives during the pandemic suggest that patients and providers view telehealth favorably [26], and telehealth has been shown to be associated with high participation rates as well as improved survival for cancer patients who smoke [27,28,29]. These suggest transitions to phone- or telehealth-based approaches may be a sustainable adjunct to increasing effective delivery smoking cessation support in cancer care. Many also reported opportunities for outreach to other clinicians and community resources, which may help bridge known barriers to communication among oncology care providers [30]. This study has significant implications related to the importance of how national coordination or prioritization might assist in maintaining access to health services in times of crisis. However, it is difficult to conceptualize a randomized approach of real-world behavioral health adaptations during a rapidly evolving healthcare crisis such as the COVID-19 pandemic. Therapeutic approaches to COVID-19 faced a similar dilemma and many studies have relied on observational cohorts. Unfortunately, observational cohort studies may imply an effect that is not accurate. It is unclear if results would be different if a smaller healthcare crisis occurred, such as a regional conflict or isolated outbreak in a single nation. Results will likely be clearer and more definitive if relevant structured research instruments were in place for immediate use when the next pandemic occurs. However, it is uncertain whether research-quality instruments would be practical or feasible while limited healthcare resources are being actively reallocated to manage massive patient influx requiring acute lifesaving care, as has occurred at multiple intervals during the pandemic. Smoking cessation has traditionally been a largely unfunded or underfunded activity in healthcare, particularly in the context of cancer care. All jurisdictions reported significant disruptions, but also reported being able to maintain or improve smoking cessation support. Results imply that the coordinated national approaches through the Network assisted in maintaining locoregional focus and performance for smoking cessation in cancer care.

## Figures and Tables

**Table 1:** Checklist used for jurisdictions’ self-assessment of behavioral counseling and pharmacotherapy implementation *.

Category	Bronze	Silver	Gold
Behavioral Counseling	Implementation of a 3A or 5A model for tobacco treatment	Advancement to an opt-out referral process to tobacco treatment	Advancement to relapse prevention and follow-up, and ability to extend support to family and friends
Pharmacotherapy	Prescription for medication or provision of nicotine replacement	Subsidized medication or nicotine replacement	Free medication or nicotine replacement

* Each jurisdiction reported the ability to provide access through a graded escalating scale of bronze, silver, or gold. Jurisdictions who had not yet achieved a bronze score were designated in a pre-implementation phase.

**Table 2:** Challenges and opportunities reported by jurisdictions during the COVID-19 pandemic.

**Challenges**	**Opportunities**
Cessation services halted, interrupted, or delayed Staff redeployed to other COVID-19 related activities or lost due to non-essential designation Reduced in-person services with shift from in-person to phone and video Decreased patient volume for cancer care Disrupted medical travel Limited activities due to quarantine Closure of community smoking cessation resources that were deemed “non-essential” Change from medication pick-up to medication delivery Change in clinical and administrative support Cancelled meetings and communications Difficulty with medical coverage or pharmacotherapy distribution Delay in approval of agreements, data requests or analysis, educational activities, and electronic resources requests Lower prioritization of smoking cessation	Shift to virtual meetings and care Increased integration and partnerships with other health groups including primary care, pharmacists, and community resources Clinical interest in smoking cessation and COVID-19 risk reduction Increased mail delivery of medications to patients Decreased interruptions and improved follow-up with patients using phone-based care Interest from new clinical groups Time to develop or refine business plans and analyze data Opportunity for increased patient education in preparation for cancer care Increased emphasis on helping staff quit smoking and improving smoke-free hospital spaces Increased financial support for medications for cancer patients Increased emphasis of smoking cessation as a standard of cancer care

**Table 3:** Timeline of program reduction challenges and recovery adaptations between March 2020 and August 2021 *.

Timeline	Mar–May 2020	Jun–Sep 2020	Oct–Dec 2020	Jan–Mar 2021	Apr–Aug 2021
Program Reduction Challenges	% of Jurisdictions				
External health system constriction Decreased program capacity including disruption of ability to maintain screening, counseling, or pharmacotherapy Loss of communication between health agencies Staff loss or redeployment Disruption of clinician or staff training Disruption of data collection or analysis Disruption of program leadership or oversight	41.7%	50%	41.7%	41.7%	8.3%
Program Recovery Adaptations	% of Jurisdictions				
Initiate revisions to budget or workplan Shift to virtual care Improved or stabilized leadership or oversight Buy-in reengaged Improved or stabilized staffing Improved delivery of smoking cessation (screening, counseling, or pharmacotherapy) Improved educational programs or materials Initiated linkage with other health providers or resources	25%	75%	58.3%	58.3%	33.3%

* Twelve jurisdictions were interviewed approximately every two to three months or as needed during the COVID-19 pandemic. Listed are challenges and adaptations reported by jurisdictions during interviews. Recorded are the percent of jurisdictions communicating one or more of the listed challenges and adaptations for the first time during each time period. While each jurisdiction could be represented in multiple time periods, each challenge or adaptation could only be reported once per jurisdiction.

## Data Availability

Not applicable.
